# ^89^Zr-labeled PSMA ligands for pharmacokinetic PET imaging and dosimetry of PSMA-617 and PSMA-I&T: a preclinical evaluation and first in man

**DOI:** 10.1007/s00259-021-05661-0

**Published:** 2021-12-21

**Authors:** Bastiaan M. Privé, Yvonne H. W. Derks, Florian Rosar, Gerben M. Franssen, Steffie M. B. Peters, Fadi Khreish, Mark Bartholomä, Stephan Maus, Martin Gotthardt, Peter Laverman, Mark W. Konijnenberg, Samer Ezziddin, James Nagarajah, Sandra Heskamp

**Affiliations:** 1 Department of Medical Imaging, Nuclear Medicine, Radboud University Medical Center, PO Box 9101, 6500 HB Nijmegen, The Netherlands; 2 Department of Nuclear Medicine, Saarland University Medical Center, Homburg, Germany

**Keywords:** Prostate cancer, PSMA-617, PSMA I&T, Radioligand therapy, Lutetium-177, Zirconium-89, Preclinical tumor model

## Abstract

**Rationale:**

Prolonged in vivo evaluation of PSMA tracers could improve tumor imaging and patient selection for ^177^Lu-PSMA-617 and ^177^Lu-PSMA-I&T. In this study, we present the radiolabeling method of PSMA-617 and PSMA-I&T with the long-lived positron emitter ^89^Zr to enable PET imaging up to 7 days post-injection. We compared the biodistribution of ^89^Zr-PSMA-617 and ^89^Zr-PSMA-I&T to those of ^177^Lu-PSMA-617 and ^177^Lu-PSMA-I&T, respectively, in a PSMA^+^ xenograft model. Moreover, we provide the first human ^89^Zr-PSMA-617 images.

**Materials and methods:**

PSMA ligands were labeled with 50-55 MBq [^89^Zr]ZrCl_4_ using a two-step labeling protocol. For biodistribution, BALB/c nude mice bearing PSMA^+^ and PSMA^−^ xenografts received 0.6 µg (0.6–1 MBq) of ^89^Zr-PSMA-617, ^89^Zr-PSMA-I&T, ^177^Lu-PSMA-617, or ^177^Lu-PSMA-I&T intravenously. Ex vivo biodistribution and PET/SPECT imaging were performed up to 168 h post-injection. Dosimetry was performed from the biodistribution data. The patient received 90.5 MBq ^89^Zr-PSMA-617 followed by PET/CT imaging.

**Results:**

^89^Zr-labeled PSMA ligands showed a comparable ex vivo biodistribution to its respective ^177^Lu-labeled counterparts with high tumor accumulation in the PSMA^+^ xenografts. However, using a dose estimation model for ^177^Lu, absorbed radiation dose in bone and kidneys differed among the ^177^Lu-PSMA and ^89^Zr-PSMA tracers. ^89^Zr-PSMA-617 PET in the first human patient showed high contrast of PSMA expressing tissues up to 48 h post-injection.

**Conclusion:**

PSMA-617 and PSMA-I&T were successfully labeled with ^89^Zr and demonstrated high uptake in PSMA^+^ xenografts, which enabled PET up to 168 h post-injection. The biodistribution of ^89^Zr-PSMA-I&T and ^89^Zr-PSMA-617 resembled that of ^177^Lu-PSMA-I&T and ^177^Lu-PSMA-617, respectively. The first patient ^89^Zr-PSMA-617 PET images were of high quality warranting further clinical investigation.

**Supplementary Information:**

The online version contains supplementary material available at 10.1007/s00259-021-05661-0.

## Introduction

Prostate cancer (PCa) is the world’s second most common malignancy in males with over 1.4 million new patients diagnosed every year. While patients in early stages of PCa show high response rates to radical treatments, the prognosis of metastasized prostate cancer (mPCa) is poor [1, 2]. Recently, prostate-specific membrane antigen (PSMA) has emerged as an ideal target for prostate cancer imaging [3, 4]. Positron emission tomography (PET) using PSMA ligands labeled with short-living positron emitters (e.g., ^68^ Ga/^18^F) is the current imaging method of choice to detect mPCa [5]. Moreover, due to the low expression of PSMA in healthy tissues, it is considered an ideal target for therapeutic applications.

In recent years, radioligand therapy with lutetium-177 (^177^Lu; a 0.5 MeV beta-emitter with a half-life of 6.7 days), targeting PSMA, became a promising new treatment for mPCa patients [6–9]. Previous studies have demonstrated that both ^177^Lu-PSMA-617 and ^177^Lu-PSMA-I&T are effective in approximately 60% of end-stage mPCa patients, while ~ 40% of patients did not respond despite high PSMA uptake on PET scans [6–10]. Therefore, it remains challenging to select patients that will best benefit from this treatment, which was also recently addressed by the authors of the pivotal trial of ^177^Lu-PSMA-617 (VISION) [11]. This is even more relevant considering the current trend of applying PSMA radioligand therapy for smaller tumors or earlier disease stages, such as hormone-sensitive PCa (NCT03828838, NCT04720157, NCT04297410, NCT04343885) [10, 12]. To date, several disease-related or patient-specific factors that could affect treatment response have been considered such as ECOG score, laboratory results, PSMA heterogenicity, [^18^F]FDG uptake, tumor localization (bone/lymph node/visceral), high versus low volume disease, and DNA-damage repair mutations [6, 8, 10, 13–20]. Yet, the heterogenous responses to this treatment remain poorly understood.

A vital aspect of systemic treatments is sufficient uptake of the cytotoxic drug into the tumorous tissue. Here, radioligand therapy has a particular advantage over other systemic therapies because of the ability to perform intra-therapeutic dose calculations using the gamma decay of the ^177^Lu ligands. This SPECT-based dosimetry could elucidate on the differential response between patients [14]. However, for ^177^Lu, this is only feasible intra-therapeutically and challenging for smaller and heterogenous tumors because of the limited resolution and sensitivity of SPECT. Thus, there is a need for new approaches to predict efficacy prior to therapy initiation and to select the most appropriate treatment dose. PET is preferred over SPECT because of its higher spatial resolution, better sensitivity, and more accurate quantification of small tumor lesions. Radionuclides such as ^68^ Ga or ^18^F cannot be used because their short half-lives do not allow to study the tumor retention of the radiotracer over a period of several days. The positron emitter ^89^Zr, with a half-life of 3.2 days, is a promising alternative frequently used for antibody imaging, including ^89^Zr-pembroluzimab, ^89^Zr-nivolumab, and ^89^Zr-trastuzumab [21]. These antibodies were conjugated with desferrioxamine (DFO) as chelator, not with DOTA or DOTAGA used in PSMA-617 and PSMA-I&T. However, DOTA labeling of ^89^Zr has recently been reported [22]. In addition to the dosimetric evaluation of PSMA tracers, we hypothesize that the later time point ^89^Zr-PSMA PET imaging could also aid in visualizing metastases that require more time for internalization of the tracer (e.g., smaller size, lower PSMA expression, low PSA values or metastases with impaired blood supply) [23] or tumors that are obscured by the high urinary uptake of PSMA tracers in the first hours (e.g., bladder uptake of [^68^ Ga]Ga-PSMA-11 or [^18^F]DCFPyL).

In the present study, we describe a novel strategy to label both a DOTA-conjugated (PSMA-617) and a DOTAGA-conjugated ligand (PSMA-I&T) with ^89^Zr. We compared the biodistribution of ^89^Zr-PSMA-617 and ^89^Zr-PSMA-I&T to those of ^177^Lu-PSMA-617 and ^177^Lu-PSMA-I&T, respectively, in PSMA^+^ tumor-bearing mice. A secondary study objective was to compare the dosimetry of ^177^Lu-PSMA-617 versus ^177^Lu-PSMA-I&T. Moreover, we present first patient images.

## Materials and methods

### Cell culture

Colon carcinoma cell line LS174T was purchased from the American Type Culture Collection. LS174T is a radiosensitive cell line with an alpha of 0.26/Gy at a low-dose rate (LDR) that has a better in vivo growth pattern compared to prostate cancer cell line LNCaP [24]. LS174T cells were stably transfected with human PSMA using the plasmid pcDNA3.1-hPSMA (PSMA^+^) and cells were tested for target expression and mycoplasma as was previously described [25]. LS174T wild-type (PSMA^−^) and PSMA^+^ cells were cultured at 5% CO_2_, 37 °C in RPMI 1640 medium supplemented with 2 mM glutamine, and 10% FCS (Life technologies). PSMA^+^ cells were cultured in the presence of 0.3 mg/ml G418 (geneticin) [25].

### *Radiolabeling PSMA-617 and PSMA I&T with [*^*89*^*Zr]ZrCl*_*4*_

First, [^89^Zr]Zr-chloride ([^89^Zr]ZrCl_4_) was produced from [^89^Zr]Zr-oxalate ([^89^Zr]Zr(ox)_2_) (PerkinElmer) according the method described by Pandya et al. with some minor modifications [22, 26]. A strong cation exchange cartridge (QMA, Waters) was activated with acetonitrile and washed with 10 mL 0.9% NaCl, 1 M HCl, and water, respectively. [^89^Zr]Zr(ox)_2_ was loaded on the activated QMA cartridge and subsequently the cartridge was washed with > 50 mL metal-free water. Trapped [^89^Zr]Zr(ox)_2_ was eluted with 0.1 M HCl in 10 fractions, 3 drops/fraction (circa 90–100 µL), with a total volume of 1 mL. All fractions were measured in a dose calibrator and the fractions with the highest [^89^Zr]ZrCl_4_ activity (fractions 7 and 8) were used for radiolabeling.

Labeling of PSMA-617 (DOTA, ABX) and PSMA I&T (DOTAGA, piCHEM) was performed in Lobind vials (Eppendorf). For the biodistribution study, 18 µg PSMA ligand (15 nmol PSMA-617, 12 nmol PSMA-I&T) was dissolved in 0.5 M MES buffer, pH 5.5, and 25 MBq [^89^Zr]ZrCl_4_ (specific activity 1.4 MBq/µg) was added. After 45-min incubation at 95 °C, the labeling reaction was stopped by adding 50 mM EDTA to complex the unlabeled [^89^Zr]Zr^4+^. To obtain a radiochemical purity of > 95% and < 3% ^89^Zr colloid content, radiolabeled ligands were purified using C18 light cartridges (Waters), eluted with 100% ethanol. The eluate was evaporated under a gentle stream of nitrogen at 95 °C and dissolved in PBS resulting in a final ethanol content < 10%.

For the PET imaging part, 10 µg PSMA ligand (8.2 nmol PSMA-617, 6.7 nmol PSMA-I&T) was dissolved in 0.5 M MES buffer, pH 5.5. Ligands were labeled with 52 MBq and 27 MBq [^89^Zr]ZrCl_4_ respectively (specific activity 5 MBq/µg for PSMA-617 and 5.1 MBq/µg for PSMA-I&T). Reactions were incubated in a two-step labeling protocol: first incubation at pH 3–4, 95 °C for 30 min, followed by addition of an extra amount of 0.5 M MES buffer (17.5 µl MES, pH 5.5), and subsequent incubation at pH 4–5 for another 30 min at 95 °C. Similar to labeling for the biodistribution study described above, labeling reaction was terminated by adding EDTA and radiolabeled ligands were purified using C18 light cartridges and dissolved in PBS.

Labeling efficiency and radiochemical purity were determined by ITLC and RP-HPLC analysis. ITLC-SG paper with 0.25 M NH_4_OAc pH 5.5/EDTA buffer as a mobile phase was used. For the determination of the ^89^Zr-colloid content, ITLC was performed on TLC-SG gel 60 RP-18 F254 aluminum sheets with 80% acetonitrile in 0.1% TFA in water as a mobile phase. HPLC analysis was performed using an Alltima C18 column (5 µm C18, 250 × 4.6 mm) at a flow rate of 1 mL/min, using 0.1% TFA in water as buffer A and 0.1% TFA in acetonitrile as buffer B in a linear gradient from 3 to 100% in 10 min. Results were quantified using GINA star software (Elysia-Raytest).

### *Radiolabeling PSMA-617 and PSMA I&T with [*^*177*^*Lu]LuCl*_*3*_

Non-carrier-added [^177^Lu]Lu-chloride ([^177^Lu]LuCl_3_) was purchased from Isotope Technologies Garching (ITG) and ready for use. For the biodistribution study, 18 µg PSMA ligand (15 nmol PSMA-617, 12 nmol PSMA-I&T) was added to 0.5 M MES buffer, pH 5.5, and 33 MBq [^177^Lu]LuCl_3_ (specific activity ^177^Lu-PSMA: 1.8 MBq/µg). After 30-min incubation at 95 °C, the labeling reaction was terminated by adding 50 mM EDTA to complex the free [^177^Lu]Lu^3+^. Labeling efficiency and radiochemical purity were determined by ITLC and RP-HPLC, similarly as used for the ^89^Zr-labeled ligands (without the ITLC for colloid determination).

For the SPECT imaging study, 5 µg PSMA ligand (4.1 nmol PSMA-617, 3.3 nmol PSMA-I&T) was added to 0.5 M MES buffer, pH 5.5, and 155 MBq [^177^Lu]LuCl_3_ (specific activity ^177^Lu-PSMA: 31 MBq/µg). After 30-min incubation at 95 °C, the labeling reaction was terminated by adding 50 mM EDTA.

### In vitro* stability*

The stability of the ^89^Zr- and ^177^Lu-labeled ligands was analyzed in PBS and human serum. The radiolabeled ligands were incubated in PBS (RT) or in human serum (37 °C) for 24 h. Aliquots were analyzed by RP-HPLC at 1, 4, and 24 h, using the HPLC protocol described above. Before analysis of the serum aliquots, serum proteins were precipitated by dilution in acetonitrile 1:1 (v/v), followed by extensive vortexing and centrifugation at 15,300 × *g* for 10 min. Supernatant of aliquots was collected and analyzed using RP-HPLC. Stability is expressed in percentage of labeled ligand.

### Animal studies

All experiments were approved by the institutional Animal Welfare Committee of the Radboud University Medical Center and were conducted in accordance to the guidelines of the Revised Dutch Act on Animal Experimentation. BALB/c nude male mice (Janvier) were housed in individually ventilated cages (Blue line IVC, 3–5 mice per cage) under nonsterile standard conditions with cage enrichment present and free access to animal chow (Sniff GmbH) and water. At 6–8 weeks of age, mice were inoculated subcutaneously with 3.0 × 10^6^ PSMA^+^ cells in the left flank and 1.5 × 10^6^ PSMA^−^ cells in the right flank, diluted in 200 μL of complete RPMI 1640 medium. Radiotracers were injected intravenously 10–14 days after tumor cell inoculation when average PSMA^+^ xenograft size was 27.6 ± 17.9 mm^3^, as determined by caliper measurements. The researchers were not blinded for the experimental groups and tumor-bearing mice were block-randomized into groups based on tumor size.

### *Pharmacokinetics *^*89*^*Zr- and *^*177*^*Lu-PSMA*

Sixteen groups of six mice bearing subcutaneous xenografts received an intravenous injection (0.6 µg ligand/mouse) of either ^89^Zr-PSMA-617 (specific activity 1.4 MBq/µg), ^89^Zr-PSMA-I&T (specific activity 1.4 MBq/µg), ^177^Lu-PSMA-617 (specific activity 1.8 MBq/µg), or ^177^Lu-PSMA-I&T (specific activity 1.8 MBq/µg) in vehicle (PBS/0.5% BSA) in the tail vein. To study the biodistribution after dissection of the four tracers over time, mice were sacrificed using CO_2_/O_2_-asphyxiation at 2 h, 1, 3, and 7 days post injection. Tumors, blood, and relevant organs and tissues were dissected, and weighed, and radioactivity in each sample as well as three corresponding activity standards (1% injection fluid) was quantified using a well-type γ-counter. The results were expressed as percentage of injected dose per gram of tissue (%ID/g).

### Biodistribution-based dosimetry

Biodistribution data obtained in the pharmacokinetic study were used to estimate the absorbed radiation doses to the organs and tumor using the 25 g mouse model in OLINDA version 2.1, with the ^89^Zr decay functioning as a dosimetry estimation model [DEM] for ^177^Lu. A one-phase exponential decay function was fitted to the activity concentration data at 2, 24, 72, and 168 h, and the cumulative activity concentration in each organ and tumor was calculated by analytic integration of the fitted expression. Absorbed radiation doses to the organs were calculated according to the MIRD scheme with the following equation: $D(rT) = \sum_{{r}_{S}}\left[\tilde{A} ({r}_{S})\right] \times {m}_{S} \times S({r}_{T} \leftarrow {r}_{S})$, with *D*(r_T_) the absorbed dose to a target organ *r*_T_, [Ã(*r*_*S*_)] the time-integrated activity concentration or total number of decays per mass in a source organ *r*_S_, and *S*(*r*_T_ ← *r*_S_) the *S*-value or absorbed dose rate to *r*_T_ per unit activity of ^177^Lu/^89^Zr in *r*_S_ with mass *m*_S_. The *S*-values and source organ masses were obtained for a standardized 25-g mouse from the RADAR realistic animal models [27]. Uncertainties in the absorbed dose values were estimated according to the EANM Uncertainty Guideline for molecular radiotherapy [28].

### PET/CT and SPECT/CT imaging

With the sole purpose to visualize the in vivo distribution of the tracers, four groups of three mice bearing subcutaneous xenografts were injected intravenously (1 µg ligand/mouse) with ^89^Zr-PSMA-617 (specific activity 5 MBq/µg), ^89^Zr-PSMA-I&T (specific activity 5 MBq/µg), ^177^Lu-PSMA-617 (specific activity 22 MBq/µg), or ^177^Lu-PSMA-I&T (specific activity 22 MBq/µg) in PBS/0.5% BSA in the tail vein. Mice were scanned under general anesthesia (2–3% isoflurane/O_2_) 2 h, 1, 3, and 7 days post injection. SPECT images (U-SPECT II, MILabs) were acquired using a 1.0-mm diameter pinhole rat collimator [29]. Mice were scanned for 20 (2 h p.i.), 30 (1 day, 3 days p.i.), or 60 (7 days p.i.) min. SPECT scans were reconstructed with MILabs reconstruction software, using an ordered-subset expectation maximization algorithm, energy windows 104.5–121.5 keV and 176.8–239.2 keV, 1 iteration, 16 subsets, and voxel size of 0.75 mm.

PET images were acquired using an Inveon animal PET scanner (Siemens Preclinical Solutions). Mice were scanned for 20 (2 h, 1 day p.i.), 30 (3 days p.i.), or 60 (7 days p.i.) min. Scans were reconstructed using Inveon Acquisition Workplace software (version 4.1, Siemens Preclinical Solutions) with iterative three-dimensional ordered subset expectation maximization using a maximum a priori algorithm with shifted Poisson distribution, with the following parameters: matrix 256 × 256 × 161, pixel size 0.4 × 0.4 × 0.8 mm, with a corresponding beta of 0.05 mm. All mice received a CT scan (spatial resolution 160 μm, 65 kV, 615 μA) for anatomical reference. Maximum intensity projections (MIPs) of the SPECT and PET images were created using the Inveon Research Workplace software version 4.1 (Siemens Preclinical Solutions).

### First human imaging

The labeling strategy of ^89^Zr-PSMA-617 and ^89^Zr-PSMA I&T was optimized warranting imaging of a prostate cancer patient. One patient with biochemical recurrence in serum prostate-specific antigen following previous radical surgery was allowed to receive PET/CT imaging with ^89^Zr-PSMA-617. In accordance with the Declaration of Helsinki, the patient provided informed consent before study entry. The patient received 90.5 MBq (corresponding 1.1 MBq/kg) of ^89^Zr-PSMA-617 followed by PET/CT imaging at 1 h, 3 h, 24 h, and 48 h post injection. PET/CT scans were performed using an EANM-accredited Biograph 40 mCT (Siemens Medical Solutions, Knoxville, TN, USA). PET data were acquired from vertex to mid-femur (4 min per bed position) and reconstructed using an iterative 3-dimensional ordered subset expectation maximization algorithm (3 iterations; 24 subsets; slice thickness 5 mm). Random correction, decay correction, scatter correction, and attenuation correction were applied. CT was performed with a low-dose technique using an X-ray tube voltage of 120 keV and tube current modulated by CARE Dose4D with a maximal tube current–time product of 30 mAs.

### Statistical analysis

Statistical analyses were performed with Graphpad Prism, version 5.03. Results are presented as mean ± SD. Differences in tumor uptake were tested for significance using a one-way ANOVA with a Bonferroni’s multiple comparison post-test. Differences were considered significant at *p* < 0.05, two-sided. Fitting of the time-activity curves was performed by least squares regression without weighting. The covariance matrix and the standard errors of the fit results were used for error analysis of the dosimetry result.

## Results

### *Labeling of PSMA-617 and PSMA-I&T with *^*89*^*Zr and *^*177*^*Lu*

PSMA ligands were labeled with [^89^Zr]ZrCl_4_, produced from [^89^Zr]Zr(ox)_2_ using a strong anion exchanger, resulting in a QMA yield of 90% [26]. Fractions 7 and 8 (containing 60% of [^89^Zr]ZrCl_4_) were used to radiolabel the PSMA ligands. Use of other fractions resulted in lower labeling efficiency (data not shown), most probably due to presence of residual oxalate anion [30].

Labeling with ^89^Zr resulted in a radiochemical yield of 90% for PSMA-617 and 93% for PSMA-I&T and a specific activity of 1.2 MBq/µg. To achieve a higher specific activity, the ^89^Zr-labeling was further optimized using a two-step procedure. PSMA ligand was incubated with [^89^Zr]ZrCl_4_ at pH 3–4 and subsequently at pH 4–5 (both 30 min, 95 °C) which resulted in a radiochemical yield of 95% and a specific activity of 5.0 MBq/µg for PSMA-617, and a radiochemical yield of 96% and a specific activity of 5.1 MBq/µg for PSMA-I&T. All ^89^Zr-labeled ligands could be purified using a C18 cartridge, resulting in a RCP of > 97% (biodistribution studies) or > 99% (PET imaging studies). ^89^Zr labeling of both ligands was stable in PBS and human serum up to 24 h (37 °C); radiochemical purity remained above 98% for all time points measured (Table 1).

**Table 1:** Stability of ^89^Zr-PSMA ligands in PBS and human serum

Time (hours)	[^89^Zr]Zr-PSMA-617		[^89^Zr]Zr-PSMA-I&T	
	**PBS (RT)**	**Serum (37 °C)**	**PBS (RT)**	**Serum (37 °C)**
0	99.7%*		99.5%*	
1	98.8%	98%	99.8%	98.9%
4	98.8%	98%	99.7%	98.3%
24	98.5%	98.7%	99.7%	99.3%

^*^Stability expressed in percentage of labeled ligand, as determined by RP-HPLC

Radiolabeling of PSMA-617 and PSMA-I&T with [^177^Lu]LuCl_3_ resulted in radiochemical yield of > 98%, a specific activity of 1.8 MBq/µg (biodistribution) and 31 MBq/µg (imaging), and a RCP > 98%.

### *Biodistribution of *^*89*^*Zr-PSMA and *^*177*^*Lu-PSMA ligands*

**Fig. 1:**
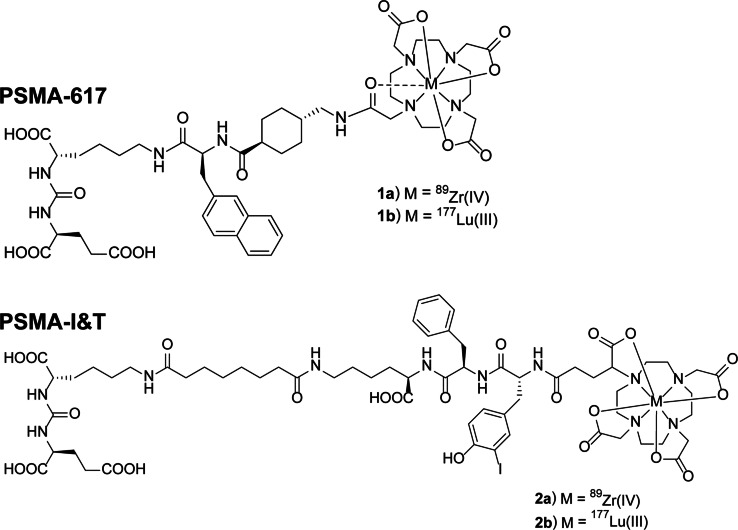
Example structures of PSMA-617 and PSMA-I&T, complexed with [^177^Lu]Lu^3+^ or [^89^Zr]Zr^4+^

**Fig. 2:**
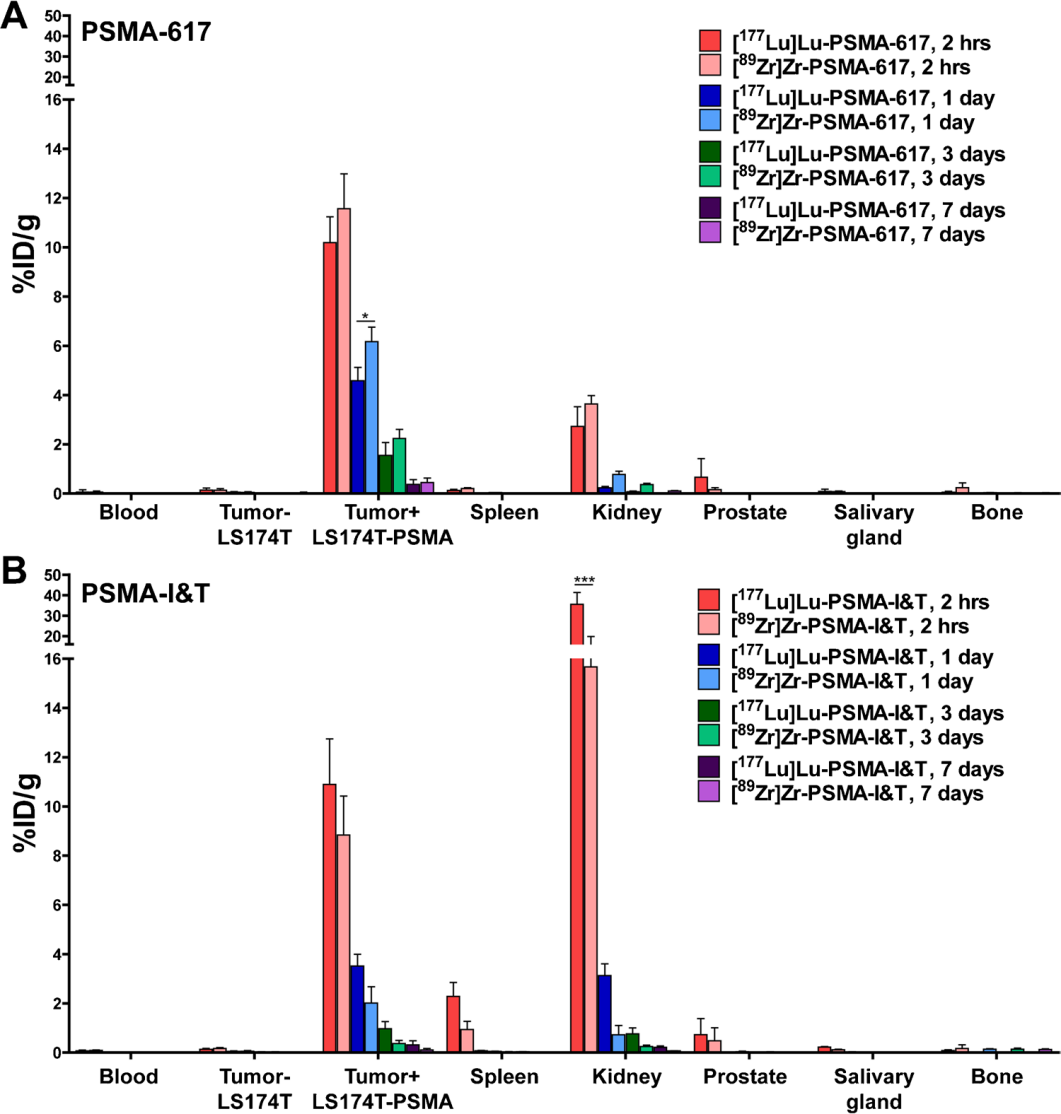
Tissue uptake of [^89^Zr]Zr-PSMA and [^177^Lu]Lu-PSMA ligands. **A** Biodistribution of 0.6 µg [^177^Lu]Lu-PSMA-617 and [^89^Zr]Zr-PSMA-617 or **B** 0.6 µg [^177^Lu]Lu-PSMA-I&T and [^89^Zr]Zr-PSMA-I&T in mice bearing subcutaneous PSMA^+^ and PSMA^−^. Biodistribution was determined after dissection at 2 h, 1 day, 3 days, and 7 days p.i. Data was corrected for decay

To evaluate whether ^89^Zr-PSMA PET-based dosimetry was similar to ^177^Lu-PSMA dosimetry, the biodistribution and pharmacokinetics of ^89^Zr- and ^177^Lu-labeled PSMA ligands were compared. Four radiotracers were assessed: ^89^Zr-PSMA-617, ^177^Lu-PSMA-617, ^89^Zr-PSMA-I&T, and ^177^Lu-PSMA-I&T (Fig. 1). All tracers showed a high and specific accumulation in the PSMA^+^ tumors, which decreased over time (Fig. 2). Minimal tracer uptake was measured in the PSMA^−^ wild-type tumor (< 0.2%ID/g) and other organs including the spleen, salivary gland, and prostate (Supplementary Tables 1 and 2).

First, a comparison was made between PSMA-617 labeled with either ^177^Lu or ^89^Zr (Fig. 2A). A similar and rapid blood clearance was observed for both tracers (Fig. 2A, Supplementary Table 1). ^177^Lu-PSMA-617 and ^89^Zr-PSMA-617 showed a mean tumor uptake of 10.2 ± 1.0 vs 11.6 ± 1.4%ID/g (2 h p.i., *p* > 0.05), respectively, which decreased at later time points. Kidney accumulation was low for PSMA-617 labeled with either ^177^Lu or ^89^Zr at all time points (< 5%ID/g). Despite the low uptake, differences in bone accumulation were observed: 0.06 ± 0.04 vs 0.24 ± 0.19%ID/g for ^177^Lu-PSMA-617 and ^89^Zr PSMA-617, respectively (2 h p.i., Supplementary Table 1).

Second, a comparison was made between ^177^Lu-labeled and ^89^Zr-labeled PSMA-I&T (Fig. 2B). A comparable rapid blood clearance was observed for both tracers (< 0.09%ID/g, 2 h p.i., Supplementary Table 2). No significant differences in tumor uptake were observed between ^177^Lu- and ^89^Zr-labeled PSMA-I&T (Fig. 2B). For example, tumor uptake of ^177^Lu-PSMA-I&T compared to ^89^Zr-PSMA-I&T was 10.9 ± 1.8 vs 8.8 ± 1.6%ID/g (2 h p.i., *p* > 0.5), respectively. Two hours p.i. kidney accumulation of ^177^Lu-PSMA-I&T was 35.6 ± 5.7%ID/g. This was significantly higher compared to ^89^Zr-PSMA-I&T, which showed a kidney accumulation of 15.7 ± 4.2%ID/g (*p* < 0.001). Bone accumulation of ^177^Lu-PSMA-I&T (0.08 ± 0.04%ID/g, 2 h p.i.) was slightly lower compared with ^89^Zr-PSMA-I&T (0.17 ± 0.15%ID/g, Supplementary Table 2).

We also compared ^177^Lu-labeled PSMA-617 and PSMA-I&T (Supplementary Tables 1 and 2). Over time, no differences in tumor uptake were observed between ^177^Lu-PSMA-617 and ^177^Lu-PSMA-I&T. Yet, kidney accumulation differed significantly between the tracers. Two hours p.i., ^177^Lu-PSMA-I&T showed a more than tenfold higher kidney accumulation of 35.6 ± 5.7%ID/g compared with 2.7 ± 0.8%ID/g for ^177^Lu-PSMA-617 (*p* < 0.001). This remained higher at all following time points.

### PET/CT and SPECT/CT imaging

**Fig. 3:**
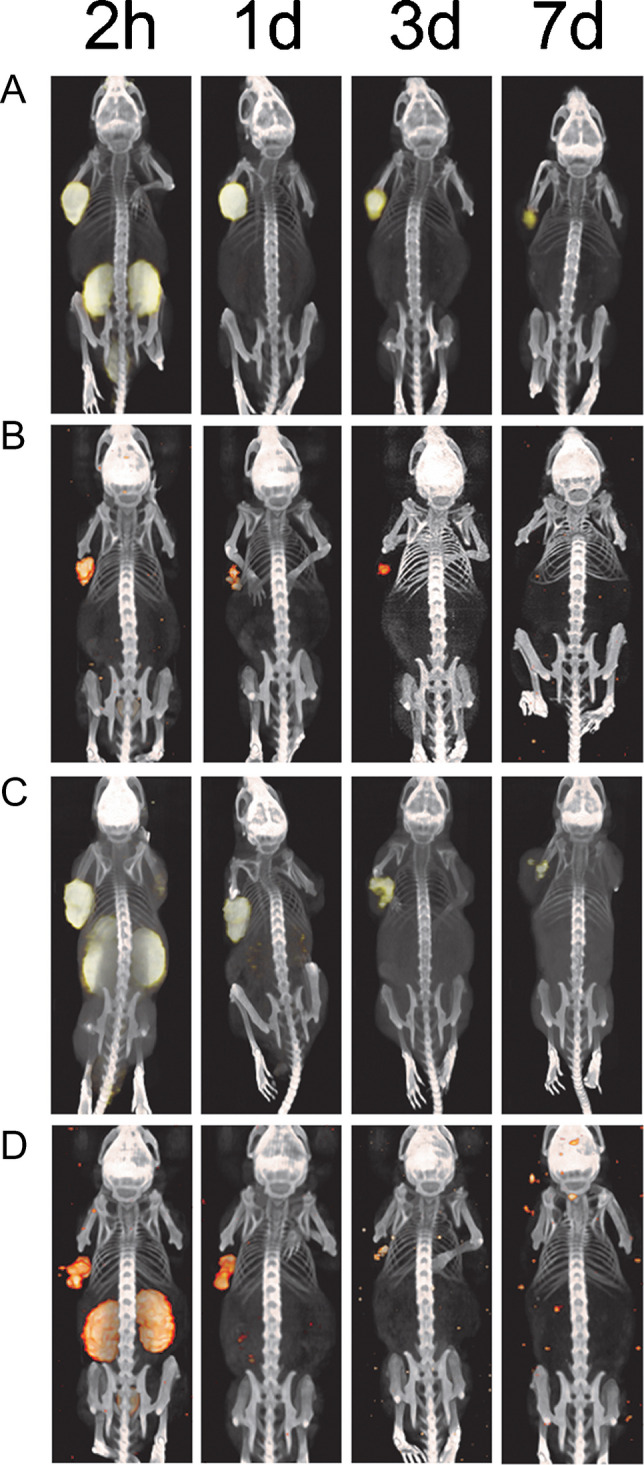
^89^Zr-PSMA PET/CT imaging and ^177^Lu-PSMA µSPECT/CT imaging of PSMA^+^ tumors. With the sole purpose to visualize the in vivo distribution of the tracers, three mice per tracer were imaged. (A) PET/CT of ^89^Zr-labeled PSMA-617 and (B) μSPECT/CT of ^177^Lu-labeled PSMA-617, (C) PET/CT scan of ^89^Zr-labeled PSMA-I&T, and (D) μSPECT/CT scan of ^177^Lu-labeled PSMA-I&T. Mice with s.c. PSMA^+^ (left flank) and wild-type PSMA^−^ (right flank), tumors were scanned 2 h, 1 day, 3 days, and 7 days p.i

The in vivo biodistribution of ^89^Zr- and ^177^Lu-labeled ligands was visualized using PET/CT or SPECT/CT imaging (Fig. 3). Mice were scanned 2 h, 1 day, 3 days, and 7 days post injection. Conforming to the biodistribution data after dissection, high PSMA-617 and PSMA-I&T tracer accumulation was observed in the PSMA^+^ xenografts (left flank) with both imaging modalities. While the PSMA^+^ tumors were clearly visible, the PSMA^−^ wild-type tumors (right flank) could not be detected. Visual assessment of the scans showed that ^89^Zr-PET scans offered similar or even higher quality imaging of the PSMA^+^ xenografts compared to the ^177^Lu-SPECT scans at all later time points (Fig. 3).

### *Dosimetry of *^*177*^*Lu-PSMA and *^*89*^*Zr-PSMA ligands*

**Fig. 4:**
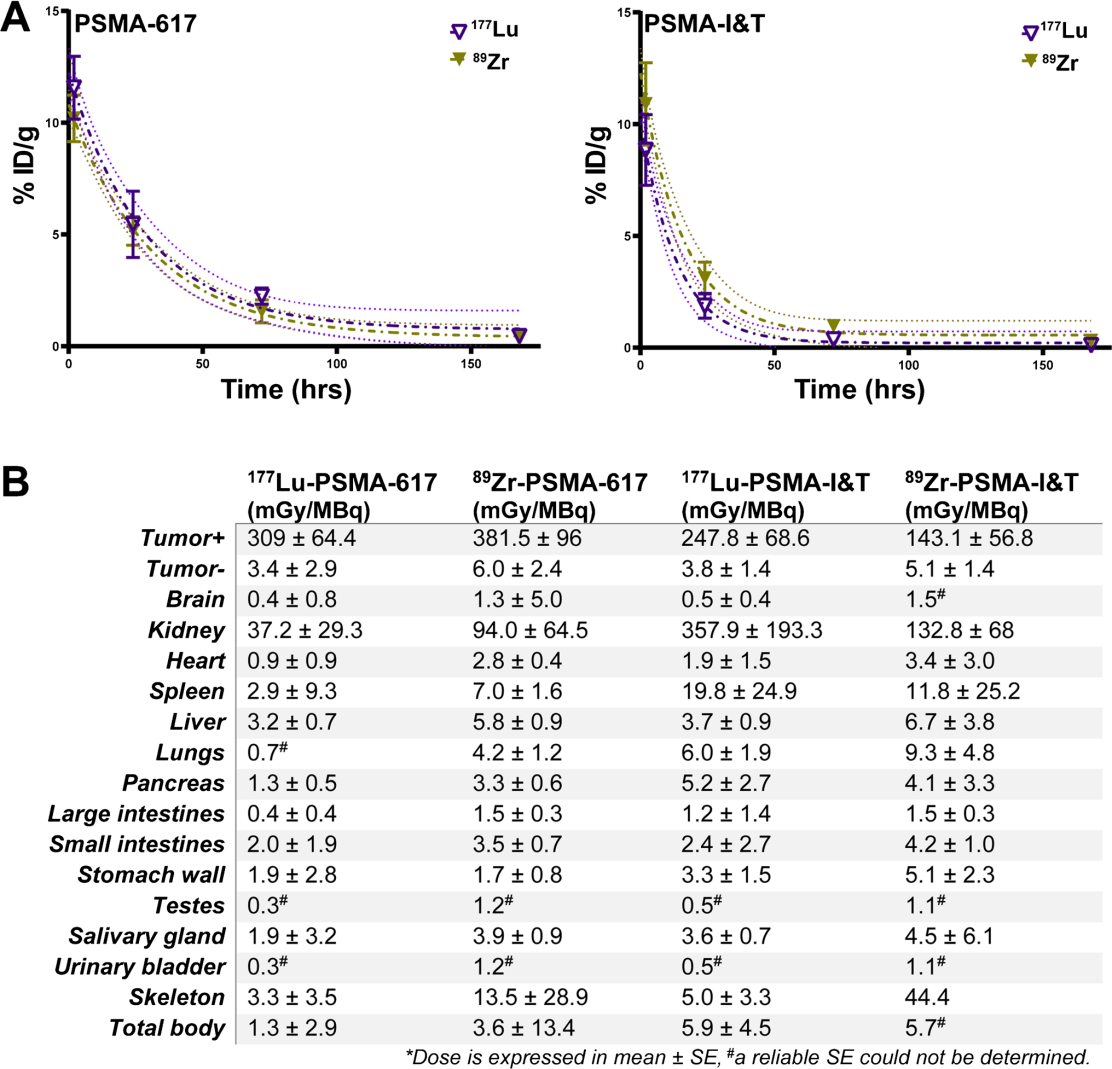
Dosimetry of ^177^Lu-labeled and ^89^Zr-labeled PSMA-617 or PSMA-I&T, based on ex vivo biodistribution. **A** Time-activity curves of ^177^Lu-labeled and ^89^Zr-labeled PSMA-617 or PSMA-I&T in the PSMA^+^ tumor. **B** Absorbed radiation doses (in mGy/MBq) of tumor and other organs were extrapolated from biodistribution data of [^177^Lu]Lu-PSMA-617, [^89^Zr]Zr-PSMA-617, [^177^Lu]Lu-PSMA-I&T, and [^89^Zr]Zr-PSMA-I&T. The ^89^Zr decay functioned as a model for ^177^Lu. Data was corrected for decay

A one-phase decay function was fitted to the biodistribution data of ^177^Lu-PSMA and ^89^Zr-PSMA at 2, 24, 72, and 168 h, to estimate absorbed radiation doses to the tumor and organs (Fig. 4A). The animal-derived dose estimations predicted that the highest absorbed radiation dose was received by the PSMA^+^ tumor followed by the kidneys (Fig. 4B). The absorbed radiation dose in the PSMA^+^ tumors for ^177^Lu-PSMA-617 was 309 ± 64 mGy/MBq, while the ^89^Zr-PSMA-617 dosimetry estimation model (DEM) predicted a ^177^Lu-PSMA-617 dose of 382 ± 96 mGy/MBq. Tumor absorbed radiation doses for ^177^Lu-PSMA-I&T and the ^89^Zr-PSMA-I&T DEM were 248 ± 69 and 143 ± 57 mGy/MBq, respectively. In accordance with the ex vivo biodistribution data, the absorbed radiation doses in the tumor did not significantly differ between ^177^Lu-PSMA and ^89^Zr-PSMA for all four tracers tested.

The absorbed radiation doses in the kidney were 37 ± 29 vs 94 ± 65 mGy/MBq for ^177^Lu-PSMA-617 and ^89^Zr-PSMA-617 DEM, respectively (*p* = 0.07). ^177^Lu-PSMA-I&T showed the highest kidney dose of 358 ± 194 mGy/MBq, which significantly differed from the ^89^Zr-PSMA-I&T DEM absorbed dose of 133 ± 68 mGy/MBq (*p* < 0.05). We calculated a higher absorbed radiation dose of ^89^Zr-PSMA-617 DEM (14 mGy/MBq) and ^89^Zr-PSMA-I&T DEM (44 mGy/MBq) by the bone as compared to that of the ^177^Lu-labeled ligands (< 5 mGy/MBq). In the other organs tested, which include the spleen, liver, and salivary gland, the absorbed doses per injected activity were below 20 mGy/MBq. In addition, total body dose was below 6 mGy/MBq and absorbed radiation doses in the PSMA-negative tumor were around 5 mGy/MBq. Figure 4B provides all dosimetry results.

## Discussion

**Fig. 5:**
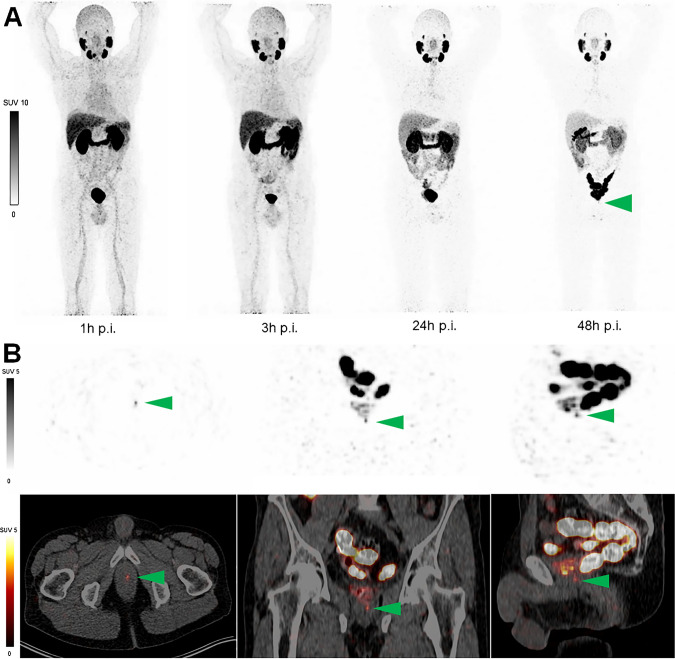
^89^Zr-PSMA-617 PET/CT images of a patient with biochemical recurrence of prostate cancer. A 64-year-old male presented with a PSA recurrence of 0.22 µg/ml and underwent PET/CT imaging after injection of 1.1 MBq/kg (total 90.5 MBq) of ^89^Zr-PSMA-617. Two years prior to the present investigational scan, he received a radical prostatectomy due to high-grade (ISUP grade 4) prostate cancer. **A** Maximum-intensity projection (MIP) after 1 h, 3 h, 24 h, and 48 h post injection of ^89^Zr-PSMA-617. **B** After 48 h post injection, a small focal lesion in the prostate bed (green arrow) was identified, which suggested a local recurrence. No other suspicious uptake was noted. The lesion could not be confirmed by histopathology due to its limited size, but was confirmed by PSA response to subsequent target external beam radiotherapy. Physiological uptake was observed in the kidney, bladder, liver, spleen, intestine, and salivary glands. CT = computed tomography; ISUP = international society of urological pathology; PET = positron emission tomography; PSA = prostate-specific antigen

Improved pre-treatment assessment of PSMA tracer pharmacokinetics is clinically relevant to further personalize radioligand therapy and improve patient selection (i.e., by doing pre-treatment dosimetry). Moreover, late time point PSMA PET imaging may aid in the detection of difficult to detect prostate cancer metastases (i.e., smaller size, lower PSMA expression, low PSA values or metastases with impaired blood supply) [23]. Due to their short half-lives, the currently available PET radiotracers (e.g., [^68^ Ga]Ga-PSMA-11 or [^18^F]PSMA-1007) are not suitable for this purpose as they only resemble the uptake at a very early time point but not the retention time of the ligand-receptor complex within the tumor cell [31]. The latter is crucial to reliably calculate the local radiation dose within the target lesion. To overcome this limitation, we have developed a radiolabeling method of PSMA-617 and PSMA-I&T with the long-lived positron emitter ^89^Zr, which enables reliable dose calculations using PET imaging. We showed that the distribution of ^89^Zr-labeled PSMA-617 and PSMA-I&T could be monitored with PET imaging and determined the potential of ^89^Zr-labeled PSMA ligands to predict the tumor and normal tissue dose of their ^177^Lu-labeled counterpart using the biodistribution data. Following these preclinical results and presented feasibility in a human patient (Fig. 5), this new compound can be readily translated into a larger clinical phase I/II study.

[^89^Zr]ZrCl_4_ production and the high-stability labeling of ^89^Zr in DOTA were previously described by Pandya et al. [22, 26]. In the current study, we further optimized this strategy for ^89^Zr-labeling of DOTA-conjugated PSMA-617 and DOTAGA-conjugated PSMA-I&T, being the optimal matching pair for PSMA-targeted diagnostics, dosimetry, and therapy. Recently, two other studies reported ^89^Zr-labeled PSMA ligands for later time point PET in prostate cancer patients [23, 32]. However, these studies used a PSMA ligand with a DFO chelator, while for ^177^Lu-PSMA-617 and ^177^Lu-PSMA-I&T a DOTA(GA) chelator is used. Alterations in the structure of the PSMA ligand, such as exchanging the chelator, can lead to changes in affinity, pharmacokinetics, and tumor uptake of these small ligands [33, 34]. Therefore, ^89^Zr-DFO-PSMA scans may not accurately reflect the biodistribution of ^177^Lu-DOTA-PSMA-617 and ^177^Lu-DOTAGA-PSMA-I&T. Furthermore, these studies did not determine how well ^89^Zr-labeled PSMA ligands could predict ^177^Lu-PSMA dosimetry for RLT. Using the two-step strategy as described in the present study, therapeutic ligands (e.g., PSMA-617 and PSMA-I&T) can be directly labeled with ^89^ZrCl_4_ with a labeling efficiency of > 95% and a specific activity of 5.0 MBq/µg and < 3% colloid. In line with our results, Imura et al. recently issued a preprint of a method to label PSMA-617 with ^89^ZrCl_4_ by using a solvent mixture of HEPES buffer and DMSO. They presented PET images at 24 h post injection of ^89^Zr-PSMA-617 of PSMA^+^ tumor in a LNCaP xenograft model [35].

The detailed biodistribution data of the present study showed that all radiolabeled PSMA ligands rapidly accumulated in the PSMA^+^ xenografts resulting in similar tumor uptake for ^177^Lu-PSMA-617 vs ^89^Zr-PSMA-617 and ^177^Lu-PSMA-I&T vs ^89^Zr-PSMA-I&T. Thus, in line with other studies investigating ^89^Zr-labeled antibodies, this indicates that ^89^Zr-PSMA PET is suitable for pre-therapeutic selection of tumors that will receive a high absorbed radiation dose from ^177^Lu-labeled PSMA [36–38]. Moreover, the first patient that received ^89^Zr-PSMA-617 PET imaging revealed a small focal lesion suggesting local recurrence on the 24 and 48 h post injection PET scan. Hence, (very) small lesions with less receptor density may need more time to internalize sufficient amounts of tracer. Therefore, ^89^Zr-PSMA PET on later time points may expose lesions that would otherwise remain unnoticed. This was also described by a recent report using ^89^Zr-DFO-PSMA [23]. Yet these observations need further evaluation in follow-up studies with histopathology as golden standard.

Ligand uptake and radiation dose of ^89^Zr- and ^177^Lu-labeled PSMA were also evaluated in healthy organs. Kidney uptake of the ^177^Lu-PSMA-I&T ligand was significantly higher compared with ^89^Zr-PSMA-I&T. This means that the kidney dose using pre-therapeutic ^89^Zr-PSMA-I&T might underestimate the kidney absorbed dose for ^177^Lu-PSMA-I&T. Based on these preclinical data, ^89^Zr-PSMA-617 followed by ^177^Lu-PSMA-617 therapy would be preferred over PSMA-I&T. However, a patient study will need to elucidate whether this difference in kidney uptake is reproducible in humans or even relevant because of the high threshold dose (40 Gy biologically effective dose) and nephrotoxicity seldomly being observed [39].

We observed a higher uptake of ^89^Zr-PSMA-617 and ^89^Zr-PSMA-I&T in the bone as compared to that of the ^177^Lu-labeled ligands. Bone uptake has been described for several ^89^Zr-labeled compounds in mice, including the radiolabeled PSMA antibody ^89^Zr-DFO-J591 [37, 40]. In these studies, the elevated bone uptake is most likely caused by instability of ^89^Zr in DFO, resulting in the release of ^89^Zr from the chelator and subsequent redistribution of weakly bound ^89^Zr to bone and cartilage (epiphysis) due to a strong affinity for phosphate [41]. However, the ^89^Zr-DOTA/DOTAGA complexation used in the present study has a superior stability [21, 22]. Still, we calculated higher doses in the bone based on ^89^Zr biodistribution data as compared with ^177^Lu biodistribution data. Bone uptake could be avoided by using a different radionuclide with less affiliation to bone, such a ^64^Cu, ^44^Sc, or ^124^I. Yet, with their half-lives of 12.7 and 4.4 h, respectively, ^64^Cu and ^44^Sc are ill-suited to determine tumor retention over the course of several days. However, successful labeling of ^64^Cu-PSMA-617 and ^64^Cu-PSMA-I&T has been described with good in vivo stability of the labeled ligand. ^64^Cu-PSMA showed high tumor uptake, but also had a predilection to the liver [42, 43]. In addition, it does not allow for later time point imaging (e.g., 5–7 days) which is crucial for reliable dosimetry as we did show earlier [18, 31]. A study is awaited that compares the biodistribution and dosimetry estimates of ^64^Cu-PSMA for ^177^Lu-PSMA-RLT. While ^124^I has a good T_1/2_ of 100.2 h, it has non-residualizing properties whereas ^177^Lu and ^89^Zr remain trapped in the cell upon internalization [44]. Therefore, ^89^Zr labeling is preferred over ^124^I. Also, in contrast to the mouse models in which high bone uptake is observed, none of the abovementioned clinical studies regarding ^89^Zr-labeled antibodies reported elevated bone uptake nor did the previous reports on ^89^Zr-DFO-PSMA [23, 32]. We also did not observe high bone uptake in the first clinical patient that received ^89^Zr-PSMA PET imaging. Thus, a clinical study in a larger cohort will need to elucidate if this impairs the imaging and dosimetry outcome of ^89^Zr-PSMA in prostate cancer (e.g., in bone metastases or bone marrow).

In addition to the comparison between ^89^Zr and ^177^Lu, we observed differences in the biodistribution of ^177^Lu-labeled PSMA-617 and PSMA-I&T. Although each group received a similar peptide and activity, we found a higher absorbed radiation dose of ^177^Lu-PSMA-I&T to the kidneys and spleen compared to ^177^Lu-PSMA-617. Recently, a preclinical comparative study of PSMA-I&T and PSMA-617 also found relatively high uptake of PSMA-I&T in the kidneys compared to the tumor, particularly in the first 8 h, similar to our study [45]. In contrast to the preclinical findings, several clinical dosimetry studies found a comparable mean absorbed radiation dose for ^177^Lu-PSMA-617 and ^177^Lu-PSMA-I&T in the kidneys [14, 46–49].

Market authorization of ^177^Lu-PSMA-617 is pending in the 3rd or 4th line castration-resistant disease with the VISION trial meeting its primary endpoints (NCT03511664) [11]. Furthermore, PSMA radioligand therapy is being evaluated in early-stage PCa patients, such as in patients with smaller lesions or (oligo)metastatic disease (NCT04343885, NCT04297410, NCT04430192, NCT04443062) [10, 12]. Especially the application in early-stage PCa patients warrants accurate pre-therapeutic PET dosimetry to improve patient selection, as these patients have a long life expectancy with several alternative treatment strategies available. Moreover, the increasing use of alpha emitters (e.g., ^225^Ac-PSMA) with their complex decay schemes, low abundance of gamma, and higher toxicity levels may also benefit from a more reliable pre-therapeutic selection model (i.e., dosimetry) than a one time point PET scan [50, 51]. While ^89^Zr-PSMA PET imaging will result in some additional radiation exposure, the extra radiation dose is of negligible amount compared to the therapy itself. Hence, the present study with ^89^Zr labeling of the two most used PSMA ligands for prediction of ^177^Lu radioligand therapy showed promising results and can be readily translated to clinical application as was shown in the first in human case (Fig. 5). We hypothesize that there are several clinical indications such as pre-PSMA radioligand therapy dosimetry and detection of difficult-to-detect metastases that may benefit from ^89^Zr-PSMA imaging. This will be evaluated in following studies.

## Conclusions

In this translational study, we present a successful labeling strategy of DOTA-PSMA-617 and DOTAGA-PSMA-I&T with ^89^Zr, the biodistribution of these novel tracers in a PSMA^+^ xenografts model, and its first clinical application in a human patient. The tumor uptake and blood clearance of ^89^Zr-PSMA-I&T and ^89^Zr-PSMA-617 resembled that of ^177^Lu-PSMA-I&T and ^177^Lu-PSMA-617, respectively. The first ^89^Zr-PSMA PET images in a patient showed high contrast of PSMA expressing tissues, also at the later time points. A patient study is planned to investigate its clinical potential.

## Supplementary Information

Below is the link to the electronic supplementary material.

Supplementary file1 (DOCX 44 KB)

## Data Availability

The datasets generated during and/or analyzed during the current study are available from the corresponding author on reasonable request.

## Abbreviations

mPCa: Metastasized prostate cancer
PCa: Prostate cancer
PSMA: Prostate-specific membrane antigen
PSMA-RLT: Prostate-specific membrane antigen radioligand therapy
PET: Positron emission tomography
^44^Sc: Scandium-44
^64^Cu: Copper-64
^89^Zr: Zirconium-89
^124^I: Iodine 124
^177^Lu: Lutetium-177
DEM: Dosimetry estimation model
