# Supplementary material for: 89Zr-labeled PSMA ligands for pharmacokinetic PET imaging and dosimetry of PSMA-617 and PSMA-I&T: a preclinical evaluation and first in man
Source: Eur J Nucl Med Mol Imaging. 2021 Dec 21;49(6):2064–76. doi: 10.1007/s00259-021-05661-0 (PMC12722385; doi:10.1007/s00259-021-05661-0)
Supplement: Supplementary file 1 — Supplementary file1 (DOCX 44 KB) [file 259_2021_5661_MOESM1_ESM.docx]

**Supplementals**

| Table S1. Biodistribution of ^177^Lu-labeled and ^89^Zr-labeled PSMA-617 (%ID/g, Mean ± SD) | | | | | | | | |
| --- | --- | --- | --- | --- | --- | --- | --- | --- |
|  | **[^177^Lu]Lu-PSMA-617** | **[^177^Lu]Lu-PSMA-617** | **[^177^Lu]Lu-PSMA-617** | **[^177^Lu]Lu-PSMA-617** | **[^89^Zr]Zr-PSMA-617** | **[^89^Zr]Zr-PSMA-617** | **[^89^Zr]Zr-PSMA-617** | **[^89^Zr]Zr-PSMA-617** |
|  | **2 hrs** | **1 day** | **3 days** | **7 days** | **2 hrs** | **1 day** | **3 days** | **7 days** |
| Biodistribution | | | | | | | | |
| Blood | 0.06±0.09 | 0.002±0.001 | 0.0001±0.0001 | 0.0004±0.0003 | 0.06±0.04 | 0.004±0.001 | 0.002±0.0004 | 0.001±0.0004 |
| Muscle | 0.04±0.03 | 0.005±0.001 | 0.0001±0.0001 | 0.001±0.001 | 0.13±0.21 | 0.009±0.002 | 0.006±0.001 | 0.004±0.001 |
| PSMA^-^ wildtype | 0.14±0.08 | 0.05±0.03 | 0.01±0.01 | 0.004±0.005 | 0.14±0.06 | 0.05±0.03 | 0.02±0.007 | 0.03±0.03 |
| PSMA^+^ | 10.19±1.04 | 4.58±0.55 | 1.56±0.52 | 0.38±0.19 | 11.57±1.41 | 6.17±0.59 | 2.24±0.36 | 0.45±0.18 |
| Heart | 0.03±0.01 | 0.005±0.002 | 0.0001±0.001 | 0.001±0.001 | 0.04±0.01 | 0.009±0.001 | 0.007±0.001 | 0.004±0.001 |
| Lung | 0.07±0.01 | 0.02±0.01 | 0.05±0.11 | 0.003±0.001 | 0.08±0.02 | 0.02±0.003 | 0.01±0.004 | 0.006±0.002 |
| Spleen | 0.13±0.04 | 0.02±0.003 | 0.006±0.005 | 0.006±0.004 | 0.21±0.03 | 0.04±0.003 | 0.03±0.004 | 0.01±0.002 |
| Pancreas | 0.03±0.01 | 0.006±0.001 | 0.002±0.001 | 0.001±0.001 | 0.04±0.01 | 0.01±0.001 | 0.006±0.001 | 0.004±0.001 |
| Liver | 0.05±0.01 | 0.02±0.003 | 0.01±0.004 | 0.01±0.004 | 0.07±0.01 | 0.03±0.001 | 0.03±0.002 | 0.02±0.002 |
| Stomach | 0.08±0.06 | 0.01±0.002 | 0.001±0.001 | 0.003±0.002 | 0.08±0.03 | 0.02±0.002 | 0.009±0.002 | 0.005±0.002 |
| Kidney | 2.73±0.80 | 0.23±0.06 | 0.08±0.02 | 0.02±0.003 | 3.64±0.34 | 0.78±0.13 | 0.36±0.05 | 0.1±0.02 |
| Adrenals | 0.24±0.10 | 0.06±0.04 | 0.00±0.00 | 0.02±0.03 | 0.52±0.16 | 0.14±0.05 | 0.13±0.11 | 0.04±0.02 |
| Duodenum | 0.07±0.05 | 0.01±0.005 | 0.005±0.01 | 0.004±0.002 | 0.07±0.01 | 0.02±0.005 | 0.01±0.005 | 0.003±0.002 |
| Prostate | 0.66±0.76 | 0.01±0.005 | 0.007±0.001 | 0.01±0.01 | 0.16±0.08 | 0.02±0.008 | 0.02±0.009 | 0.01±0.004 |
| Salivary glands | 0.09±0.09 | 0.01±0.001 | 0.007±0.006 | 0.002±0.002 | 0.08±0.02 | 0.03±0.006 | 0.01±0.001 | 0.006±0.002 |
| Bone marrow | 0.20±0.13 | 0.10±0.02 | 0.00±0.00 | 0.04±0.02 | 0.64±0.18 | 0.23±0.11 | 0.19±0.07 | 0.07±0.04 |
| Bone | 0.06±0.04 | 0.02±0.005 | 0.003±0.003 | 0.005±0.005 | 0.24±0.19 | 0.04±0.006 | 0.03±0.006 | 0.03±0.006 |
| Ratio’s | | | | | | | | |
| Tumor to salivary gland | 181±92 | 147±34 | 375±56 | 262±59 | 220±139 | 175±31 | 150±115 | 96±93 |
| Tumor to spleen | 88±27 | 56±7 | 224±44 | 146±16 | 224±86 | 92±19 | 66±51 | 42±15 |
| Tumor to kidney | 3.97±1.08 | 3.19±0.35 | 20.96±5.28 | 8.13±1.62 | 20.60±6.75 | 6.17±0.66 | 19.39±9.8 | 5.07±3.47 |

Data was corrected for decay

| Table S2. Biodistribution of ^177^Lu-labeled and ^89^Zr-labeled PSMA-I&T (%ID/g Mean ± SD) | | | | | | | | |
| --- | --- | --- | --- | --- | --- | --- | --- | --- |
|  | **[^177^Lu]Lu-PSMA-I&T** | **[^177^Lu]Lu-PSMA-I&T** | **[^177^Lu]Lu-PSMA-I&T** | **[^177^Lu]Lu-PSMA-I&T** | **[^89^Zr]Zr-PSMA-I&T** | **[^89^Zr]Zr-PSMA-I&T** | **[^89^Zr]Zr-PSMA-I&T** | **[^89^Zr]Zr-PSMA-I&T** |
|  | **2 hrs** | **1 day** | **3 days** | **7 days** | **2 hrs** | **1 day** | **3 days** | **7 days** |
| Biodistribution | | | | | | | | |
| Blood | 0.07±0.03 | 0.004±0.001 | 0.001±0.001 | 0.001±0.001 | 0.09±0.02 | 0.01±0.002 | 0.002±0.001 | 0.001±0.001 |
| Muscle | 0.04±0.02 | 0.01±0.001 | 0.002±0.002 | 0.001±0.001 | 0.07±0.05 | 0.01±0.001 | 0.01±0.002 | 0.005±0.001 |
| PSMA^-^ wildtype | 0.14±0.04 | 0.05±0.02 | 0.02±0.02 | 0.003±0.002 | 0.17±0.04 | 0.06±0.03 | 0.02±0.01 | 0.01±0.009 |
| PSMA^+^ | 10.90±1.85 | 3.52±0.48 | 0.97±0.29 | 0.31±0.17 | 8.84±1.58 | 3.01±0.67 | 0.37±0.13 | 0.11±0.06 |
| Heart | 0.08±0.03 | 0.01±0.002 | 0.004±0.002 | 0.002±0.001 | 0.06±0.01 | 0.01±0.002 | 0.01±0.001 | 0.008±0.001 |
| Lung | 0.32±0.07 | 0.04±0.005 | 0.03±0.003 | 0.02±0.001 | 0.27±0.05 | 0.04±0.01 | 0.03±0.007 | 0.01±0.003 |
| Spleen | 2.29±0.56 | 0.08±0.01 | 0.03±0.008 | 0.02±0.006 | 0.94±0.34 | 0.05±0.008 | 0.04±0.009 | 0.02±0.005 |
| Pancreas | 0.21±0.03 | 0.01±0.003 | 0.003±0.001 | 0.001±0.001 | 0.12±0.02 | 0.01±0.001 | 0.01±0.002 | 0.006±0.002 |
| Liver | 0.07±0.02 | 0.02±0.005 | 0.01±0.002 | 0.01±0.005 | 0.07±0.01 | 0.03±0.005 | 0.03±0.003 | 0.02±0.003 |
| Stomach | 0.13±0.03 | 0.02±0.005 | 0.004±0.003 | 0.002±0.001 | 0.14±0.03 | 0.02±0.006 | 0.01±0.003 | 0.01±0.004 |
| Kidney | 35.57±5.71 | 3.13±0.48 | 0.76±0.24 | 0.22±0.06 | 15.67±4.18 | 0.72±0.38 | 0.24±0.06 | 0.07±0.007 |
| Adrenals | 2.40±0.87 | 0.36±0.07 | 0.03±0.06 | 0.04±0.04 | 1.68±0.37 | 0.23±0.09 | 0.16±0.09 | 0.06±0.02 |
| Duodenum | 0.10±0.06 | 0.02±0.003 | 0.003±0.002 | 0.004±0.004 | 0.09±0.01 | 0.02±0.002 | 0.01±0.005 | 0.01±0.005 |
| Prostate | 0.73±0.66 | 0.02±0.006 | 0.003±0.003 | 0.005±0.008 | 0.48±0.53 | 0.04±0.02 | 0.03±0.01 | 0.02±0.01 |
| Salivary glands | 0.22±0.02 | 0.03±0.006 | 0.01±0.003 | 0.006±0.003 | 0.12±0.03 | 0.02±0.003 | 0.02±0.004 | 0.009±0.003 |
| Bone marrow | 0.24±0.09 | 0.14±0.08 | 0.06±0.07 | 0.01±0.03 | 0.35±0.19 | 0.34±0.18 | 0.65±0.67 | 0.10±0.03 |
| Bone | 0.08±0.04 | 0.02±0.01 | 0.006±0.004 | 0.004±0.005 | 0.17±0.15 | 0.14±0.02 | 0.14±0.04 | 0.13±0.02 |
| Ratio’s | | | | | | | | |
| Tumor to salivary gland | 49±8.5 | 78.5±12.4 | 133.8±26.3 | 103±28.5 | 87.7±16.6 | 24.2±9.9 | 45.8±25.1 | 13±89.5 |
| Tumor to spleen | 5.2±2.4 | 10.4±4.0 | 47.7±10.6 | 38.7±11.6 | 32.8±13.9 | 10.2±2.8 | 23.9±12.1 | 7.5±5.3 |
| Tumor to kidney | 0.31±0.07 | 0.59±0.16 | 1.14±0.21 | 3.06±1.14 | 1.30±0.32 | 1.55±0.60 | 1.44±0.78 | 1.58±0.91 |

Data was corrected for decay
